# Fosthiazate inhibits root-knot disease and alters rhizosphere microbiome of *Cucumis melo* var. *saccharinus*

**DOI:** 10.3389/fmicb.2022.1084010

**Published:** 2023-01-06

**Authors:** Huifang Wang, Wanrong Yan, Jiguang Luo, Xiangping Zeng, Zhixiang Zhao, Xiaoli Dou, Meiying Fu

**Affiliations:** ^1^Key Laboratory of Plant Diseases and Pests of Hainan Province, Research Center of Quality Safety and Standards for Agro-Products, Institute of Plant Protection, Hainan Academy of Agricultural Sciences, Scientific Observation and Experiment Station of Crop Pests in Haikou, Ministry of Agriculture and Rural Affairs, Haikou, China; ^2^Key Laboratory of Green Prevention and control of Tropical Plant Diseases and Pests, College of protection, Ministry of Education, Hainan University, Haikou, China

**Keywords:** *Cucumis melo* var. *saccharinus*, fosthiazate, root-knot disease, Meloidogyne, bacterial diversity, metabolomic profiling

## Abstract

Root-knot nematodes especially Meloidogyne spp. are considered as most destructive obligate parasites that substantially reduce crop yield and quality. Fosthiazate is an efficient organothiophosphate chemical with nematicidal activity against Meloidogyne spp. The present study aimed to analyze the efficacy of fosthiazate against root-knot disease in *Cucumis melo* var. *saccharinus* and its potential effects on rhizosphere microbiome and metabolites. The fosthiazate (40%) was applied two times by spraying on the day of transplanting and during the pollination period (after 31 days). Samples from treatment (fosthiazate 40%: MF) and control groups (untreated plants; MCK) were analysed through metagenomic and metabolomic profiling of rhizospheres. Results revealed that root-knot index of the MF group (9.26 ± 1.28) was significantly (*p* < 0.05) lower than the MCK group (22.06 ± 0.71) with a control effect of 57.85% after 31 days of the first spray, whereas fosthiazate efficacy reduced to 31.87% after 38 days of second application with significantly (*p* < 0.05) different root-knot index values (MF: 56 ± 1.43 and; MCK: 82.26 ± 3.87). However, *Cucumis melo* var. *saccharinus* fruit yield in both groups (MCK: 21.1 ± 0.9 and MF: 21.53 ± 0.85) showed no differences (*p* > 0.05). Metagenomic profiling revealed Proteobacteria, Acidobacteriota, and Firmicutes as predominant phyla and Bacillus, Sphingomonas, and Acidibacter as predominant genera in rhizosphere soil samples of both MF and MCK groups. Further, a *t*-test revealed higher differential enrichment of Firmicutes at phylum level and Bacillus at genus level in MF than MCK. Metabolomic profiling of rhizospheric soil revealed a total of six differential metabolites (*p* < 0.05), four of them (Sucrose, Hexaonic acid 1, (Z)-9-Octadecenamide 1, and Hexadecanamide) were up-regulated in MF group, whereas two of them (2,3,4-Trihydroxy-3-(Hydroxymethyl) Butanol and Sulfurous acid, 2, ethylhexylundecyl ester) were down-regulated in CK group. Our study concluded that fosthiazate exhibits a better control over the rook-knot disease in the short term and resulted in trackable changes in rhizosphere microbiome and metabolome.

## Introduction

Melon (*Cucumis melo L.*) is an important cash crop that belongs to the family Cucurbitaceae, which contains 1,000 species such as watermelon, cucumber, squash, and pumpkins (Salehi et al., 2021). It is widely cultivated all over the world from temperate, tropical, and subtropical climates to arid regions (FAO, 2017). China is the main melon-producing country followed by the United States, Turkey, Iran, and India (Al-Daghari et al., 2020). Globally, the area under melon cultivation was around 1,039,691 ha with a total production of 27,501,360 tons in 2019 (Faostat, 2019). In China, it is a key economic fruit crop which is cultivated over 376, 000 ha and produces more than 13 million metric tons fruit annually, with half of the melon production in the Hainan Province, China (Yang et al., 2019; Li et al., 2021).

Root-knot nematodes are thought to be to most destructive obligate parasites in agriculture, causing reduced crop quality and production (Yigezu Wendimu, 2021). Nematodes infestation leads to an adverse economic loss of about 125 billion dollars every year around the world, accounting for approximately 14% of crop production losses (Tapia-Vázquez et al., 2022). Meloidogyne is an economically important genus comprised of various phytoparasitic nematode species that can damage nearly all vascular plants through disrupting root system, i.e., gall development (Escobar et al., 2015). Meloidogyne-infected plants show stunted growth, leaves chlorosis, wilting, and even plant death in worse scenarios. As a result, host plants become more susceptible to the invasion of various other bacterial and fungal pathogens (Ralmi et al., 2016). It covers more than 3,000 species of host plants globally (Moens et al., 2009). *M*. *incognita*, *M*. *enterolobii*, *M*. *hapla*, *M*. *arenaria*, and *M*. *javanica* are some crucial Meloidogyne species that can be found in Africa, Asia, northern United States, Europe, Caribbean, and Oceania (Coyne et al., 2018). The circumstances necessary for cantaloupe production are remarkably similar to those that encourage nematode activity (Freckman and Caswell, 1985). Studies revealed that the occurrence of muskmelon Meloidogyne is a serious threat in Wenchang City of Hainan Province (Zhourong et al., 2021). It is an emerging consensus that the outbreak of pests and diseases is because of harsh agronomic practices such as continuous cropping. Continuous cropping is the planting of the same crop over the years in the same cultivation area (Gao et al., 2019). Meanwhile, it could change physicochemical properties as well as the microbial communities of soil. As a result, agricultural crops could be threatened in terms of growth and production. Furthermore, it may gradually reduce the beneficial soil microorganisms and promote pathogenic microbes leading to the aggravation of pests over time (Pervaiz et al., 2020).

Nematicides are chemicals used to enhance crop yield by controlling the pest population. Fosthiazate, fluopyram, and avermectin are commonly available nematicides in China (Yue et al., 2020). Among them, fosthiazate is an efficient organothiophosphate chemical that has nematicidal activity against Meloidogyne (Li et al., 2020). It impairs nematode synapses ability to function normally, which in turn minimizes the level of root invasion (Huang et al., 2016). The interaction between RKN and the rhizosphere microbial communities in the soil ecosystem has not been explored extensively. Soil rhizosphere microbial communities play a significant role in regulating various biological activities such as improved plant growth, nutrition, and production, pest/ disease suppression, and ability to withstand adverse climate conditions (Qu et al., 2020). Moreover, they also enhance various abiotic indicators of soil quality including crop residue degradation, nitrogen fixation and mineralization, and soil aggregation (Saleem et al., 2019). Hence, the impact of soil microbial communities must be addressed to evaluate the plant response in diverse environmental conditions. Metagenomic sequencing emerged as a comprehensive genomic tool to evaluate the microbiome and its complex interactions in the rhizospheric zone of the plant (Priya et al., 2021). Furthermore, metabolomic profiling has been extensively used to simultaneously detect several endogenous metabolites produced by the rhizosphere microbiome (van Dam and Bouwmeester, 2016). Rhizosphere metabolome carries not only plant-secreted metabolites but also microbial secretions along with decomposed products of organic matter (Zhu et al., 2016). Recently, the 16S rDNA and high-throughput metagenomic sequencing have been exploited the potential to explore the rhizosphere bacterial community in several crops (Xu et al., 2019).

The occurrence of Meloidogyne in *Cucumis melo* var. *saccharinus* (Hami melon) is serious in Hainan Province, China. Fosthiazate is currently the most widely used pesticide in the production of *Cucumis melo* var. *saccharinus* in Hainan, and it is considered more effective against Meloidogyne in *Cucumis melo* var. *saccharinus*. This study was designed to examine the efficacy of fosthiazate against root-knot nematodes (Meloidogyne) of *Cucumis melo* var. *saccharinus* and to explore rhizosphere microbiome and metabolomic differences between the fosthiazate sprayed area and the blank control area, to understand its impact on the metabolome and microbiome of *Cucumis melo* var. *saccharinus*. These findings will provide the basis for the development of prevention and control technology.

## Materials and methods

### Experimental site location and soil preparation

The experiment was carried out in Xiatongtian Village, Huaqiao Farm, Dongfang City, Hainan Province, China (N: 108.680129; E: 18.987616). The experimental site was sandy loam soil with moderate organic matter content. The experimental field (of *Cucumis melo* var. *saccharinus*) was irrigated with spray belts, with a moderate planting density (about 3,000 plants/mu), and the management of each experimental plot was uniform. Root irrigation was performed on the day of transplanting (March 18, 2022). The experiment was designed with two treatments, one with fosthiazate nematicide (40%) application (labeled as MF) to *Cucumis melo* var. *saccharinus* plant for prevention against Meloidogyne root-knot disease and the second was control group (labeled as MCK) without any nematicide treatment.

### Fosthiazate drug application and efficacy test

The test drug contained 40% thiazophosphine water solution prepared by Hebei Sannong Agricultural Chemical Co., Ltd. China. The drug was applied two times by spraying on the day of transplanting (March 18, 2022) and during the pollination period (April 18, 2022) at a dosage of 400 ml/mu (mu = 666.7 m^2^). A completely randomized design (CRD) was applied with two treatments and each treatment had four replications (control group, MCK: MCK1, MCK2, MCK3, and MCK4, and; 40% thiazophosphine, MF: MF1, MF2, MF3, and MF4). Each replication plot area was 20m^2^. A diagonal five-point sampling method was adopted (five plants were selected from each replication). No nematicides were used within 1 month before the experiment and the results of this experiment were not affected by the previous drug use. Further, no other drugs were used to control root-knot nematode disease during the test. Throughout the experimental period, weather remianed normal and greenhouse facility was not affected by rainfall. The occurrence of root-knot disease in the roots of *Cucumis melo* var. *saccharinus* was investigated twice (April 18, 2022, and May 26, 2022), and root knot index was determined. The present investigation was carried out following the national standard “Field Efficacy Test of Nematicides for Controlling Root Nematodes.” The following formulas were used for the calculation of the root-knot disease index and control efficasy.


$$\begin{array}{c} \mathrm{Root knot}\\ \mathrm{index} \end{array}=\frac{\sum \;\left(\begin{array}{l} \mathrm{The number of diseased plants}\;\mathrm{at}\;\\ \mathrm{all}\;\mathrm{levels}\times \mathrm{disease progression} \end{array}\right)}{\mathrm{The total number of plants investigated}\times 9}\times 100$$

Root knot control effect(%)=Root knot index of control area−Root knot index of treatment areaRoot knot index of control area×100

During the whole experiment, the crops in each treatment grew normally, and no phytotoxicity was observed.

Further, the plants were graded two times (March 18, 2022 and May 26, 2022) by observing the root system (Level 0: no root-knot in the root system; Grade 1: The root system has only a few root knots; Grade 3: The root-knot is obvious, and the root-knot percentage is less than 25%; Grade 5: The root-knot percentage is 25 to 50%; Grade 7: The root-knot percentage is 50 to 75%; Grade 9: The root-knot percentage is above 75%). Grading first time, an average of 15 plants were selected from each treatment (MCK and MF), whereas 22 plants from the control group (MCK) and 20 plants from fosthiazate treatment (MF) area were selected for second time grading. The yield of *Cucumis melo* var. *saccharinus* was measured by taking the 10 random fruit samples from each replicate.

### Soil sampling for metagenomic analysis

For the metagenomic analysis, completely randomized design (CRD) was applied with two treatments MCK (control group) and MF (40% fosthiazate) repeated five times (MCK: MCK1, MCK2, MCK3, MCK4, and MCK5 and; MF: MF1, MF2, MF3, MF4, and MF5) respectively. Five-point sampling method was adopted (five plants from each replicate). The soil was dug around the roots and loosely attached soil with roots was collected into ziplock bags (Smalla et al., 1993). The samples from each replicate were combined and five biological samples were taken for repeated determination. The samples were brought to labs and passed through a 2 mm sieve to remove impurities and then stored at -80°C to be used later for DNA extraction and metagenomic sequencing.

### DNA extraction, library preparation, and metagenomic sequencing

DNA was extracted from rhizosphere samples using the Cetyl trimethyl ammonium bromide (CTAB) method (Fu et al., 2017). The DNA quantification was done by using the NanoDrop 2000-UV spectrophotometer (Thermo Fisher Scientific, Waltham, MA, United States), and DNA quality was analyzed by running on 1% agarose gel. The genomic DNA was diluted to 1 ng/ μl. The diluted DNA as a template, V4 region primers (515F and 806R) specific to the 16S rDNA gene for the detection of bacterial communities. The Phusion® High-Fidelity PCR Master Mix with GC buffer (New England Biolabs, United Kingdom), and enzymes with high-efficiency and high-reliability were used to ensure accurate and effective PCR amplification. Additionally, amplification of 16S (V4–V5, V8) archaeal-bacterial regions was performed. The PCR products were detected on 2% agarose gel followed by the analysis of PCR products using the purification kit (GeneJET Gel Extraction Kit by Thermo Fisher’s Scientific, United States). A total of 1 μg from the PCR product was used for the construction of the genomic library using the TruSeq® DNA PCR-Free Sample Preparation Kit library builder kit following the manufacturer’s guidelines. The constructed library was quantified by Qubit and qPCR. After the quantification, NovaSeq6000 was used for sequencing.

### Metagenomic sequence analysis

Quality control of sequence data was performed by splicing out the raw data (low-quality reads) using the Qiime (V1.9.1)^1^ software to obtain high-quality clean reads. High-quality reads were further filtered out and then chimeric sequences were identified using the annotation database^2^ and removed by using the UCHIME algorithm in USEARCH (Edgar et al., 2011). All clean data (effective reads) were then clustered at 97% similarity into operational taxonomic units (OTUs; Wang et al., 2007) using the Uparse algorithm (Uparse v7.0.1001)^3^ followed by the selection and screening of the representative sequences having the highest frequency in OTUs. Common and unique OTUs were presented through a Venn diagram. For the diversity and richness analysis, the dilution curves and Rank abundance curves were plotted using R software (Version 4.1.2). Species annotation analysis of OTU sequences was conducted at various taxonomic levels (kingdom, phylum, class, order, family, genus, and species) through the SSUrRNA database of SILVA138.1^4^ (July 16, 2022) set as threshold of 0.8–1. The MAFFT (v7.490^5^, July 06, 2022) software was used for prompt multiple sequence alignment (MSA) to obtain all OTUs signifying the phylogentic relationship of all sequences (Sokol et al., 2022). Moreover, the data from each sample was normalized in comparison with the samples containing minimum data quantity in order to get standardized results. After the process of data normalization, alpha diversity analysis (Observed_OTUs, Chao1, ACE, Shannon, and Simpson) was performed using the Qiime software (Version 1.9.1) and beta diversity Principal coordinate analysis (PCoA) was performed using the R software (Version 4.1.2). LEfSe analysis was performed for the identification of biomarker taxa in both groups using LEfSe software (set threshold LDA score of 4). Further in Metastats analysis, paramutation tests in both groups were conducted using Mothur software to find the value of ps. Subsequently, the obtained value of p was revised using the Benjamini and Hochberg False Discovery Rate methods to get q-value (White et al., 2009).

### Non-targeted metabolomic analysis

For the metabolomic analysis, a total of 10 biological rhizosphere samples, 5 from the control group (CK) and 5 from the treatment group (Fos), were collected and labeled as CK1, CK2, CK3, CK4, and CK5 and Fos1, Fos2, Fos3, Fos4, and Fos5, respectively. These samples were stored at -80°C and then crushed to powdered form at room temperature when needed for DNA extraction. For each sample, 0.5 g were taken and 1 ml of methanol: isopropanol: water (3:2:2) mixture was added followed by shaking at room temperature, and then kept in the ice water bath for 20 min. Centrifugation was done at 12,000 rpm for 3 min at 4°C and the supernatant was transferred to an injection bottle. An internal standard of 0.020 ml (10 μg/ml), followed by drying out with nitrogen and then freeze-dried in a lyophilizer. Then 0.1 ml of methoxyamine salt pyridine (0.015 g/ml) was added and an oxidation reaction in an oven at 37°C for 2 h was carried out with the subsequent addition of 0.1 ml BSTDS at 37°C for 37 min to obtain derivatization solution. This solution was diluted to 1 ml, filtered, and stored at 20°C. Further, GC–MS analysis was performed to evaluate the 5 samples from each group (CK and Fos).

### GC–MS analysis

The Agilent 8,890 gas chromatograph coupled to a 5977B mass spectrometer with a DB-5MS column (30 m length × 0.25 mm i.d. × 0.25 μm film thickness, J&W Scientific, United States) was employed for GC–MS analysis of the samples. Helium as carrier gas was used at a 1.2 ml/min flow rate. Injections with a split ratio of 5:1 and volume of 1 μl were obtained in front of inlet mode. The temperature of the oven fluctuated from 40°C for 1 min, and then raised to 100°C at 20°C/min and 300°C at 15°C/min, and then maintained at 300°C for 5 min. All rhizosphere samples were examined in scan mode. The temperature of the ion source and transfer line was 230°C and 280°C, respectively.

Unsupervised Principle component analysis (PCA) was performed through the statistics procomp function in the R software package. Hierarchical cluster analysis (HCA) for both rhizosphere samples and metabolites was conducted and results were presented as heatmaps. Differential metabolites for both group samples were identified by VIP score (VIP ≥ 1) and absolute Log_2_FC (Log_2_FC ≥ 1). VIP values were obtained from OPLS-DA results using the R package MetaboAnalystR containing score and paramutation plots. Before OPLS-DA, the data were log-transformed (log_2_) as well as mean-centered. Subsequently, a paramutation test was carried out to standardize the OPLS-DA model, reported in previous studies (Swenson et al., 2015). KEGG Compound database^6^ was used for the annotation of enriched metabolites followed by the mapping of annotated metabolites to identify metabolite pathways using the KEGG Pathway database^7^ (Marco-Ramell et al., 2018).

## Results

### Drug efficacy results

The nematicidal efficacy of fosthiazate was evaluated against root-knot nematodes through determining root-knot index and effective control for each treatment of *Cucumis melo* var. *saccharinus* (Table 1). Compared to the control (22.06 ± 0.71), fosthiazate treatment (9.26 ± 1.28) showed a significant (*p* < 0.05) decrease in the root-knot index with a control effect of 57.85% after 31 days of the first application. The drug efficacy (control effect) of fosthiazate was reduced to 31.87% after 38 days of second application as the root-knot index was increased in the fosthiazate treatment group (56 ± 1.43) but it was still significantly (*p* < 0.05) lower than control (82.26 ± 3.87). It showed that effective control of fosthiazate against root-knot nematodes decreased after a longer duration. Further, results revealed no significant (*p* > 0.05) differences in fruit yield in both groups (MCK vs. MF) as shown in Table 1.

**Table 1:** The efficacy of nematicide (fosthiazate 40%) after 31 and 38 days of first and second spraying, respectively against the meloidogyne and yield of *Cucumis melo* var. *saccharinus* fruit.

Treatment	After 31 days of first spraying		After 38 days of second spraying		
	Root-knot index	Effective control (%)	Root-knot index	Effective control (%)	Yield/10 cantaloupes (Kg)
MCK	22.06 ± 0.35^a^	–	82.26 ± 1.94^a^	–	21.10 ± 0.45
MF	9.26 ± 0.64^b^	57.85	56 ± 0.72^b^	31.87	21.53 ± 0.43

Means ± SEM values are given for each treatment with four replicates. Values in the same row with different letters (a and b) differ significantly (*p* < 0.05).

Further, plants of both experimental groups (MCK and MF) were graded (grade 1–9) by observing the percentage of root-knot disease in the root system of *Cucumis* (Table 2). Grading results after 31 days of first fosthiazate spray revealed that 55 ± 6.38% of observed plants in MCK group had grade 1 disease following the second highest in grade 3 (36.67 ± 1.92%), whereas 53.33 ± 4.71% of observed plants had no disease in root system (Level 0) in the MF following second highest in grade 1 (31.67 ± 5.00%). None of the observed plants had grade 7 and grade 9 disease in both groups. After 38 days of the second application of fosthiazate, the percentage of observed plants for MCK groups was highest in grade 9 (56.54 ± 2.05%) with the second highest in grade 7 (22.25 ± 2.43%), whereas the fosthiazate treated group (MF) had the highest observed plants in grade 5 (33.67 ± 3.10%) with second highest in grade 7 (20.86 ± 1.71%). Comparatively, the fosthiazate disease control effect was higher in the first 30 days of transplanting than in the second half after the second application.

**Table 2:** Root-knot disease graded (1–9) in the root system of *Cucumis melo* var. *saccharinus* treatment groups (MCK and MF).

After 31 days of the first spray						
Treatment	Level 0	Grade 1	Grade 3	Grade 5	Grade 7	Grade 9
MCK	1.67 ± 1.67^b^	55 ± 6.38^a^	36.67 ± 1.92^a^	6.67 ± 0.00^a^	0	0
MF	53.33 ± 4.71^a^	31.67 ± 5.00^b^	15 ± 3.19^b^	1.67 ± 1.67^b^	0	0
**After 38 days of the second spray**						
MCK	0	4.29 ± 1.65^b^	6.58 ± 2.24	10.33 ± 1.62^b^	22.2 ± 2.43	56.54 ± 2.05^a^
MF	0	17.29 ± 1.04^a^	13.55 ± 2.12	33.67 ± 3.10^a^	20.86 ± 1.71	14.63 ± 1.32^b^

The values are in percent (%) of observed plants in the given grade. Means ± SEM values are given for each treatment with four replicates. Values in the same row with different letters (a and b) differ significantly (*p* < 0.05).

### Operational taxonomic units and annotation results of bacterial communities

High-grade rDNA amplicons were subjected to the NovaSeq6000 sequencing platform to unravel the core bacterial communities in the *Cucumis melo* var. *saccharinus* rhizosphere. In total, 847,832 raw tags (with an average of 84,743 tags per sample) and 820,557 clean tags (with an average of 82,056 tags per sample) after quality control were produced in both MCK and MF groups. These tags were then clustered into a total of 7,892 OTUs observed at different taxonomic levels (from phylum to species) of *Cucumis melo* var. *saccharinus* rhizosphere samples using the Silva database (release132). Moreover, 6,128 (77.64%) OTUs were found to be common between MCK and MF, whereas 900 and 864 OTUs were unique in MCK and MF, respectively (Supplementary Figure S1A). Further, the refraction and rank abundance curves were drawn that revealed the number of sequences were enough in each sample (replicate) to evaluate the bacterial communities in MCK and MF (Supplementary Figures S2A,B). Additionally, the refraction curve indicated that each sample had equally diverse bacterial communities corresponding to an increase in the number of sequences (Supplementary Figure S2A). Similarly, the rank abundance curve exhibited the relative species abundance starting from the most abundant in MCK4 to the least abundant in the MF5 group (Supplementary Figure S2B).

### Alpha diversity

The alpha diversity analysis was conducted by employing five diversity indices (Observed _OTUs, Chao1, ACE, Shannon, and Simpson) to evaluate the differences in bacterial communities between MCK and MF (Table 3). The observed OTUs, Chao 1, and ACE indices revealed no significant differences (*p* > 0.05) between MCK and MF, whereas the Shannon and Simpson indices revealed significant differences (*p* < 0.05) in bacterial communities between MCK and MF.

**Table 3:** The estimation of alpha diversity indices of bacterial communities of MCK and MF samples of *Cucumis melo* var. *saccharinus* rhizosphere.

Alpha diversity parameters	Control group (MCK)	Fosthiazate treated group (MF)	*p* value
Observed_OTUs	4,543 ± 51	4,365 ± 114	0.1959
Shannon	10.17 ± 0.04	9.99 ± 0.04	0.0148
Simpson	0.9978 ± 0.20	0.9970 ± 0.00	0.0039
Chao1	5,425 ± 50	5,203 ± 122	0.131
ACE	5,564 ± 57	5,338 ± 135	0.162

Means ± SEM values are given for each treatment with five replicates.

### Differential bacterial communities

**Figure 1:**
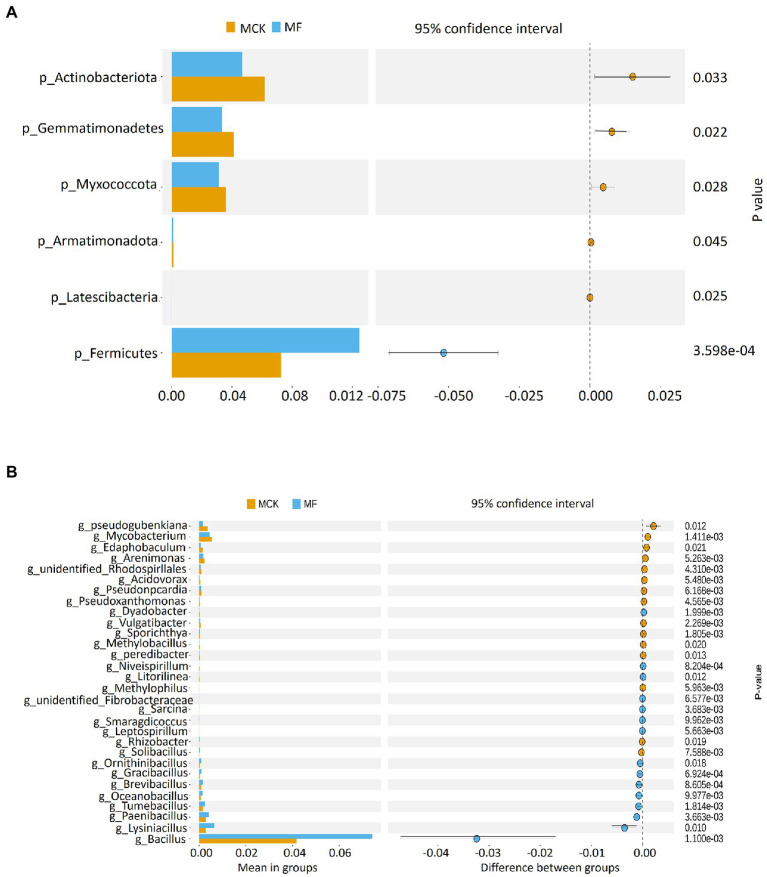
*T*-test showing bacterial groups with significant differences at **(A)** phylum and **(B)** genus levels between *Cucumis melo* var. *saccharinus* treatment groups MCK and MF at *p* < 0.05.

The relative abundance of top 10 bacterial phyla and genera were used for the identification of the predominant phylum and genus structure in both MCK and MF (Supplementary Figures S3A,B). The top three prevailing bacterial phyla colonizing the rhizosphere were Proteobacteria, Acidobateriota, and Firmicutes (Supplementary Figure S3A). The top 10 bacterial phyla accounted for about 90% of total bacterial communities. Among these, the proteobacteria were more prevalent in the rhizospheric bacterial communities with an average abundance of 40.75 and 38.93% in MCK and MF, respectively. The top three predominant bacterial genera were Bacillus, Sphingomonas, and Acidibacter in MCK and MF (Supplementary Figure S3B). The relative abudance of the Bacillus genus was high in MF (7.41%) than in MCK (4.17%). Furthermore, a *t*-test (*p* < 0.05) was performed to identify the highly significant bacterial communities at phylum and genus levels between MCK and MF (Figures 1A,B). At the phylum level, Firmicutes were highly abundant (*p* < 0.05) in MF (Figure 1A). While at the genus level, Bacillus was the most abundant genus (*p* < 0.05) in MF (Figure 1B). Additionally, a heatmap of 30 genera was constructed to classify the significantly enriched genera in both MCK and MF (Supplementary Figure S4). A heatmap chart revealed that the bacterial genera such as Pseudomonas, Bryobacter, Hyphomicrobium, and Sphingomonas were highly enriched in MF, whereas the bacterial genera such as Streptomyces, Gemmatimonas, Steroidobacter, and Micromonospora were highly enriched in MCK group.

**Figure 2:**
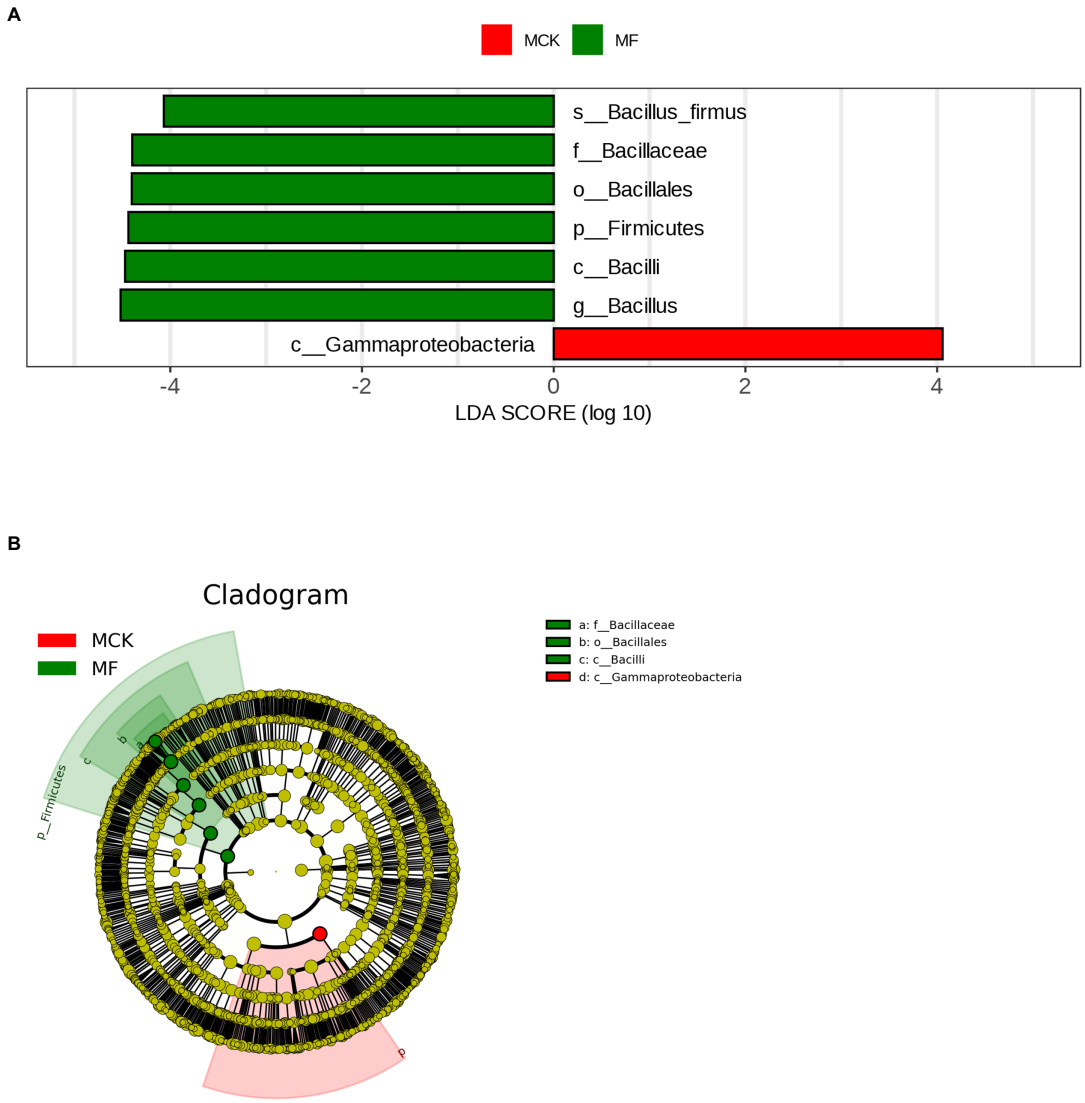
**(A)** Histogram shown by LEfSe for the computations of LDA score (LDA score ≥ 4) for dominant bacterial taxa (biomarker) between control group (MCK) and treatment group (MF) of *Cucumis melo* var. *saccharinus.* **(B)** Cladogram demonstrating the significantly dominant bacterial taxa for both MCK and MF at each taxonomic level.

The LEfSe analysis (LDA score ≥ 4) was carried out to explore the high-dimensional biomarker in different bacterial communities at various taxonomic levels in MCK and MF (Figure 2A). Gammaproteobacteria was found as biomarker taxa at the class level in MCK, whereas 6 bacterial taxa Bacillus firmus, Baciliaceae, Bacillales, Firmicutes, Bacilli, and Bacillus were found as biomarker taxa at specie, family, order, phylum, class, and genus level, respectively in MF. Furthermore, the top genera attained from multiple sequence alignment and relative abundance analysis were used to develop LEfSe Cladogram that presented the phylogenetic relationship of rhizospheric bacterial communities in both MCK and MF (Figure 2B).

### Beta diversity

**Figure 3:**
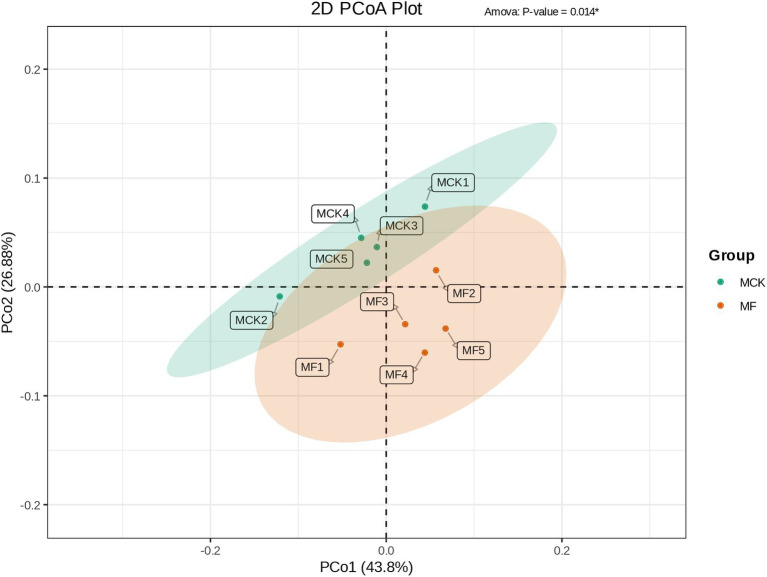
PCoA based on the bacterial composition showing the distribution of each group samples and differences between the groups (MCK and MF).

Principal coordinate analysis (PCoA) was carried out to evaluate the differences and similarities in bacterial communities between MCK and MF groups (Figure 3A). The PCo1 and PCo2 explained 43.80 and 26.88% of the total variation, respectively. The MCK group samples (MCK1, MCK2, MCK3, MCK4. and MCK5) displayed more similarities (less diversity) in bacterial communities than the MF group samples (MF1, MF2, MF3, MF4, and MF5). PCoA revealed that MCK and MF groups were segregated apart which indicated that there were substantial differences in bacterial composition between both groups (Figure 3).

### Differential metabolite analysis

**Figure 4:**
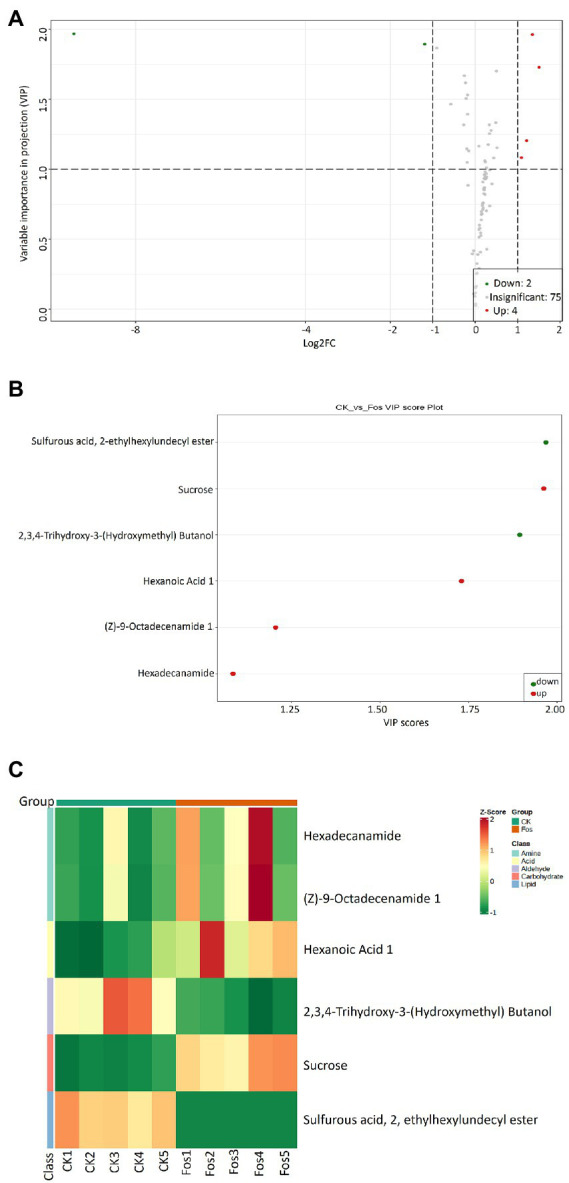
**(A)** Variable importance in projection (VIP) plot indicating the importance of each variable in CK and Fos groups. Variables with VIP score > 1 are significantly different and up regulated in CK while variables with VIP score < 1 are down-regulated in Fos. **(B)** VIP plot showing the differential expression of significantly important metabolites in both groups (Ck vs. Fos). Each dot is representing a specific metabolite; the red dot is showing the significantly up regulated metabolites while the green dot showing the significantly down-regulated metabolites. **(C)** Heatmap showing the differential microbial metabolites of *Cucumis melo* var. *saccharinus* rhizosphere samples, control group samples (CK1, CK2, CK3, CK4, and CK5) and treatment group samples (Fos1, Fos2, Fos3, Fos4, and Fos5).

PCA was carried out to analyze the similarities and differences between the metabolomic samples of the control group (CK) and the fosthiazate treated group (Fos) of *Cucumis melo* var. *saccharinus* (Supplementary Figure S5). The PC1 and PC2 revealed 56.38 and 17.48% of the total variance, respectively. The 2D PCA plot showed that CK and Fos had different metabolites as their samples were separated apart. Further, Variable importance in projection (VIP) plot was made to show the differential metabolites of *Cucumis melo* var. *saccharinus* treatment groups (CK vs. Fos) (Figures 4A,B). A total of 81 differential metabolites were identified, among them, 75 metabolites were non-significant (*p* > 0.05) and 6 metabolites were significant (*p* < 0.05) in CK vs. Fos (Figure 4). Out of 6 significant metabolites, 4 metabolites were up-regulated (VIP ≥ 1) while 2 were down-regulated (VIP ≤ 1). These 4 up-regulated metabolites were Sucrose, Hexaonic acid 1, (Z)-9-Octadecenamide 1, and Hexadecanamide, whereas 2 down-regulated metabolites were 2,3,4-Trihydroxy-3-(Hydroxymethyl) Butanol and Sulfurous acid, 2, ethylhexylundecyl ester (Figures 4B). Additionally, a heatmap was plotted to examine the enrichment of these metabolites in CK and Fos (Figure 4C). All the four up-regulated metabolites (Sucrose, Hexaonic acid 1, (Z)-9-Octadecenamide 1, and Hexadecanamide) were found highly enriched in Fos, whereas the two down-regulated metabolites (2,3,4-Trihydroxy-3-(Hydroxymethyl) Butanol and Sulfurous acid, 2, ethylhexylundecyl ester) were found highly enriched in CK.

### KEGG annotation

KEGG analysis showed three highly enriched KEGG pathways in CK vs. Fos (Supplementary Figure S6). The metabolism category was found highly enriched with Starch and sucrose metabolism, Metabolic pathways, Galactose metabolism, and Biosynthesis of secondary metabolites pathways followed by organismal systems (taste transduction and carbohydrate digestion and absorption) and environmental information processing (Phosphotransferase system and ABC transporters) in CK and Fos.

## Discussion

### Nimaticidal efficacy of fosthiazate

Root-knot nematodes particularly Meloidogyne spp. are deleterious soil-borne pathogens that cause huge economic losses of $157 billion in the agriculture sector, globally (Abad et al., 2008). Their control is quite difficult because of their ability to inhabit plant roots (Radwan et al., 2012). Currently, many chemical nematicides are used to control root-knot nematodes. In preceding reports, fosthiazate has been used to control root-knot disease in cucumber resulting in a reduced root-gall index and increased crop yield in the nematicide treatment group compared with the untreated control group (Toth et al., 2019). Further, fosthiazate in combination with other nematicides has reduced root galling activity of *Meloidogyne icognita* and also increased tomato yield (Landi et al., 2018). In the present experiment, fosthiazate was applied two times, once at the time of transplanting and second at the time of pollination after 31 days against root-knot nematode (Meloidogyne) of *Cucumis melo* var. *saccharinus* to evaluate its nematicidal efficacy. Fosthiazate reduced the root-knot index in MF compared to MCK after both applications but control effect (57.85%) achieved after first application was higher (31.87%) than second application. It revealed that time duration after application of fosthiazate reduced the drug efficacy against rook-knot nematodes. Previous studies also revealed decrease in root-knot index in fosthiazate treated plants (Landi et al., 2018; Toth et al., 2019). Further, the disease severity (grade 1–9) was higher in MCK than MF but fruit yield of *Cucumis melo* var. *saccharinus* had no significant differences between fosthiazate treated group and control group. However, previous studies revelead that fruit yield was increased with nematicidal use due to control of disease spread (Pofu and Mashela, 2022). One possible reason for these findings might be the dvelopement of resistance to fosthiazate as it has been used repeatedly in China to control nematodes (Huang et al., 2016), as in our experiment after second application of fosthiazate the drug control efficacy was decreased and disease spread was increased with higher number of plants in grade 5 and 7, especially at the time of fruit development and growth. The other possible reason might be the limited supply of water for development of fruit, as drip irrigation system was used in this experiement and due to sandy loam mostly water supply is limited to root system than other plant parts (Abdelkhalik et al., 2019). The fosthiazate druge efficacy results showed that the nematicide drug had better control in short term than long term. These findings indicate that time interval for fosthiazate application is important to achieve sustained control over nematodes.

### Metagenomic and metabolomic profile of bacterial communities *Cucumis melo* var. *saccharinus* rhizosphere

The rhizosphere plays an essential role in the association between the host plant and the soil microbial community (Priyadharsini et al., 2016). Plant-microbe association is influenced by various factors such as root exudates (Narula et al., 2012), soil properties (Kuramae et al., 2012), climate effects (Jin et al., 2018), biotic stresses including several diseases and pests (Benitez et al., 2017), plant species, plant growth stages, and roots phenotype (de la Fuente Cantó et al., 2020). As a result, rhizosphere microbes; known as plant growth-beneficial rhizosphere microorganisms (PGBR) regulate plant growth and their ability to induce disease resistance (Harish et al., 2019). A major challenge in microbial ecology is the development of strategies for efficiently manipulating and engineering beneficial plant-associated microbiomes. Recent study showed that the use of biochar in soil indirectly reduced the cadmium uptake by promoting the PGBR in rhizosphere soil (Zhou et al., 2022). Similarly, the use of rhizosphere microbiome transplant (RMT) from resistant Solanaceae eggplant varieties against pathogen *Ralstonia solanacearum* to susceptible model Solanaceae tomato variety promoted the plant growth and resulted in reduction of disease incidence rate (Jiang et al., 2022). Previous studies described that the use of nematicides can decrease the root knot disease index (Landi et al., 2018; Toth et al., 2019), which can indirectly promote the plant growth and development due to less disease burden, hence this could positively effect the microbiome and metabolome profile of plant rhizosphere (Huang et al., 2021). In the present study, the alpha diversity (Shannon and Simpson) and beta diversity indices revealed significant differences between the rhizosphere bacterial communities of *Cucumis melo* var. *saccharinus* treatment groups (control group: MCK and fosthiazate treated group: MF). Further, it also indicated that MF group had more diverse bacterial communities than MCK group. Furthermore, Firmicutes at phylum level and Bacillus spp. at genus level were found highely abundant in MF than MCK. In previous studies, high abudance of Firmicutes was associated with root-knot disease suppression in tobacco (Huang et al., 2020). These bacteria are involed in production of various lytic enzymes and antimicrobial components that confer nematicial activity against nematodes (Zhou et al., 2019). Previous studies also suggested the high abundance of Bacillus genus in samples treated with a mixture of pesticide and fertilizer in Zhanjiang, China (Huang et al., 2021). The Bacillus genus helps plants in nutrients acquisition either directly or indirectly, protects the plant from pathogen attack, or improves plant growth *via* production of phytohormones (Saxena et al., 2020). In addition, the high abundance of Bacillus was found negatively correlated with disease incidence from RKN (Zhou et al., 2019). LefSe analysis documented Gammaproteobacteria as biomarker taxa in the MCK and Fermicutes and Bacillus as biomarker taxa in MF. Studies revealed the disease-suppressive activity of Gammaproteobacteria against root-knot nematodes (Zhou et al., 2019). The diversity of Gammaproteobacteria was considered a potential plant health indicator (Köberl et al., 2017). Moreover, out of 6 significant metabolities, 4 metabolites (Sucrose, Hexaonic acid, (Z)-9-Octadecenamide, and Hexadecanamide) were up-regulated in Fosthiazate treated group (Fos), whereas 2 metabolites (Sulfurous acid, 2, ethylhexylundecyl ester and 2,3,4-Trihydroxy-3-(Hydroxymethyl)Butanol) were down-regulated in control group (CK). Sucrose is a dominant photosynthate that is produced by plants either for their metabolic activities or released into root exudates important for host colonization by rhizomicrobes (Etesami and Adl, 2020). Sucrose is involved in the acquisition of plant nutrients (carbon source) for microbial growth that results in altered microbial community structure for biocontrol activities (Tian et al., 2022). Hexatonic acid is another metabolite possessing antagonism activity against various biotic stresses such as bacteria and nematodes (Wang et al., 2019). Octadecenamide possess antimicrobial, anti-inflammatory, and antioxidant activities (Nxumalo et al., 2020). Hexadecanamide is a fatty acid amide that plays an important role in plant-microbe interaction and plant physiology (Sun et al., 2016). Further studies have shown that Hydroxymethyl exhibited strong herbicidal activity against *Amaranthus hybridus* (Adetunji et al., 2019). Our findings revealed that fosthiazate treated group had more diverse bacterial communities and positively upregulated metabolites enrichment than control group indicating that fosthiazate controlled the root-knot disease well, which indirectly led to increased plant growth and development and positively effected the rhizosphere metagenome and metabolome profile. Thus nematicidal effect of fosthiazate was potentiated by its positive effect achieved through increased abundance of soil bacteria against phytopathogens.

## Conclusion

Our study concluded that fosthiazate (40%) had a greater control effect in the short-term period than longer durations against root-knot nematodes (*Meloidogyne* spp.) of *Cucumis melo* var. *saccharinus*. At this concentration, no drug toxicity was detected. Further, the fosthizate application suppressed the disease and resulted in trackable changes in metagenomic and metabolomic profiles.

## Data availability statement

The datasets presented in this study can be found in online repositories. The names of the repository/repositories and accession number(s) can be found in the article/Supplementary material.

## Author contributions

HW and MF: conceptualization. HW, WY, JL, and XZ: data curation. HW, WY, and JL: formal analysis. MF: funding acquisition, project administration, supervision, and writing-review and editing. HW, XZ, ZZ, and XD: investigation and methodology. ZZ and XD: resources. HW: validation and writing-original draft. HW and WY: visualization. All authors have read and approved the final version of this manuscript.

## Funding

This work was supported by China Agriculture Research System of MOF and MARA (CARS-16-E18) and Hainan Province Science and Technology Special Fund (ZDKJ202002).

## Conflict of interest

The authors declare that the research was conducted in the absence of any commercial or financial relationships that could be construed as a potential conflict of interest.

## Publisher’s note

All claims expressed in this article are solely those of the authors and do not necessarily represent those of their affiliated organizations, or those of the publisher, the editors and the reviewers. Any product that may be evaluated in this article, or claim that may be made by its manufacturer, is not guaranteed or endorsed by the publisher.

## Supplementary material

The Supplementary material for this article can be found online at: https://www.frontiersin.org/articles/10.3389/fmicb.2022.1084010/full#supplementary-material

## Footnotes

^1^ http://qiime.org/scripts/split_libraries_fastq.html

^2^ https://github.com/torognes/vsearch/

^3^ http://www.drive5.com/uparse/

^4^ http://www.arb-silva.de/

^5^ https://mafft.cbrc.jp/alignment/software/

^6^ http://www.kegg.jp/kegg/compound/

^7^ http://www.kegg.jp/kegg/pathway.html
