# Supplementary material for: Fosthiazate inhibits root-knot disease and alters rhizosphere microbiome of Cucumis melo var. saccharinus
Source: Front Microbiol. 2023 Jan 6;13:1084010. doi: 10.3389/fmicb.2022.1084010 (PMC9853079; doi:10.3389/fmicb.2022.1084010)
Supplement: Supplementary file 1 [file Data_Sheet_1.docx]

**Supplementary material**

**
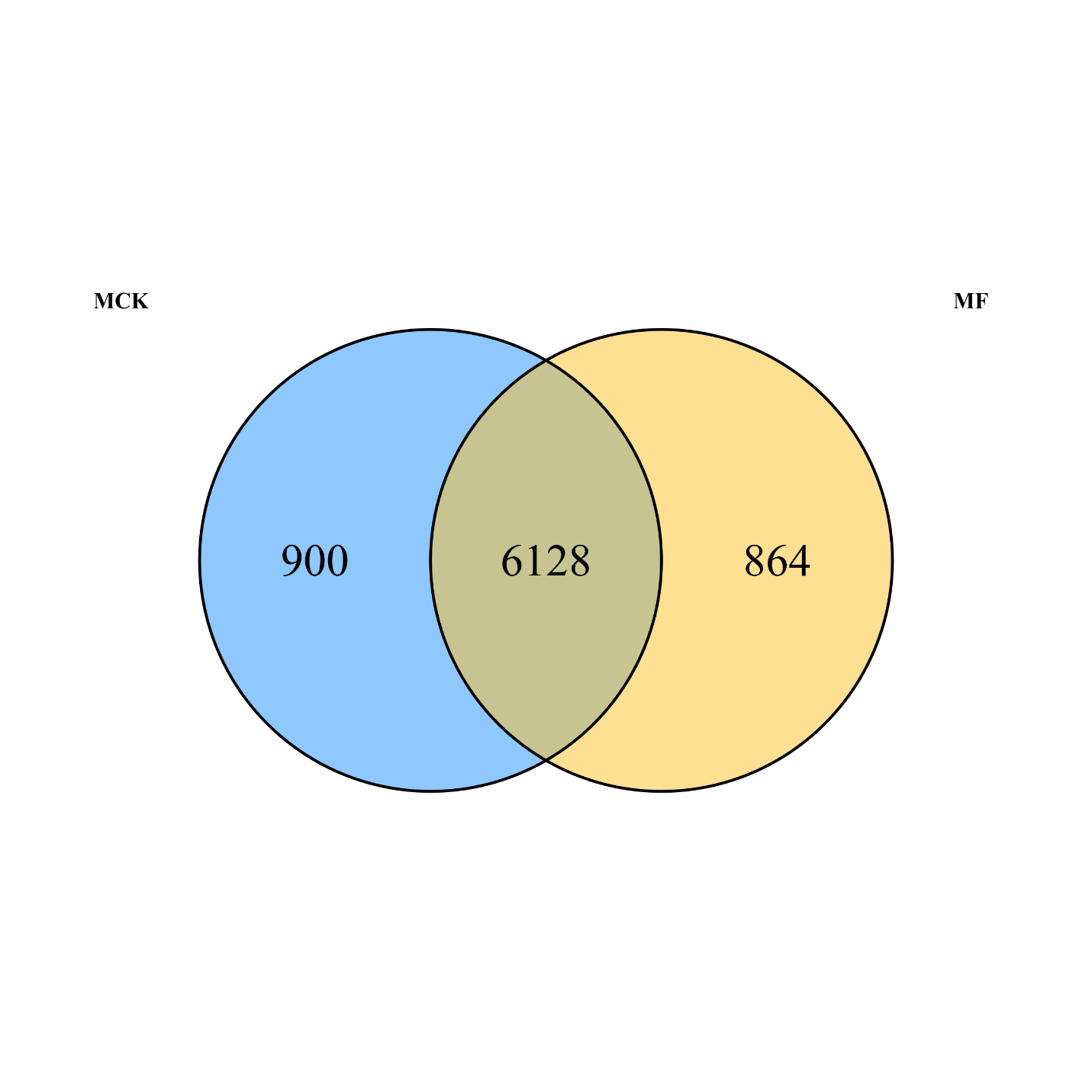
**

**Supplementary Figure S1.** Venn diagram showing the common and unique OTUs between rhizosphere samples of *Cucumis melo var. saccharinus*  treatment groups (control group: MCK and fosthiazate treated group:MF).

**
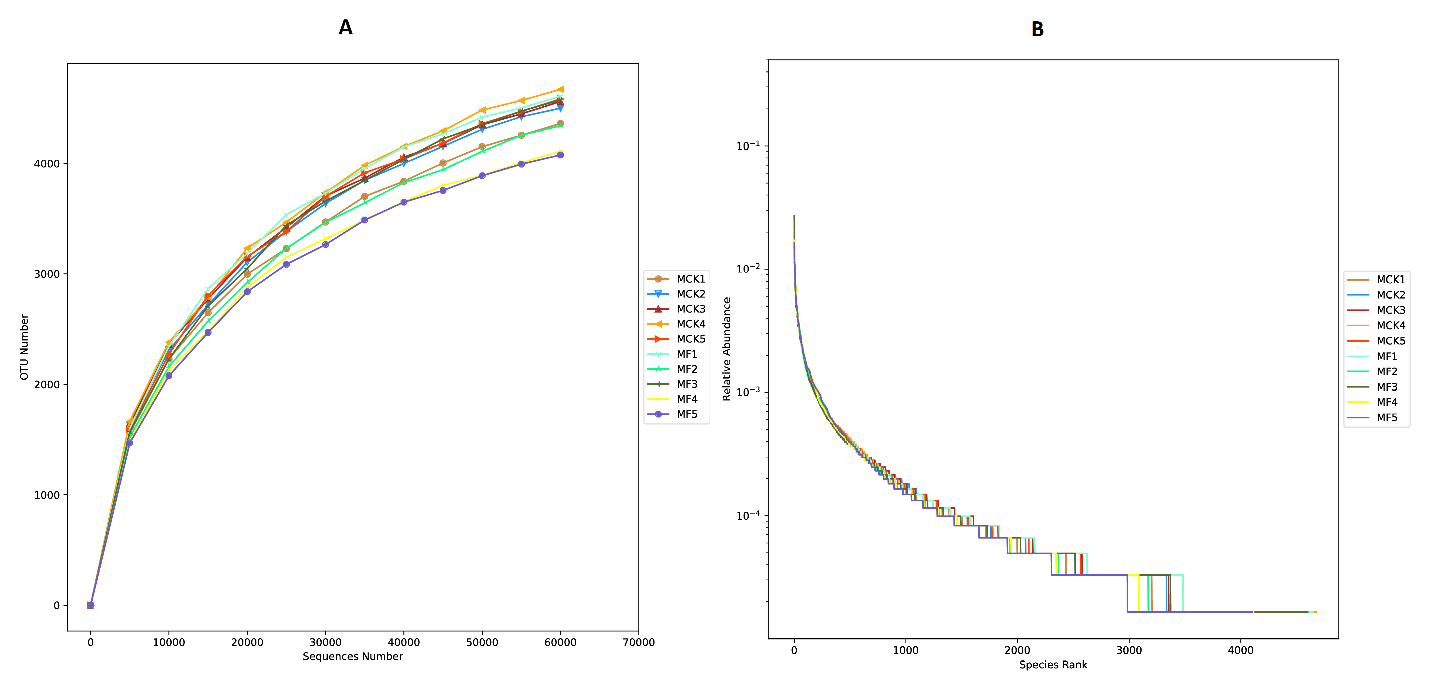
** **Supplementary Figure S2A,B.** **(A)** Rarefaction curve showing the observed number of OTUs (Operational Taxonomic Units) with respect to the number of sequencing bars drawn randomly for each sample of *Cucumis melo var. saccharinus* treatment groups (control group: MCK and fosthiazate treated group: MF) **(B)** Rank Abundance curve showing the relative abundance and species rank for each sample of *Cucumis melo var. saccharinus* treatment groups (control group: MCK and fosthiazate treated group: MF).

**
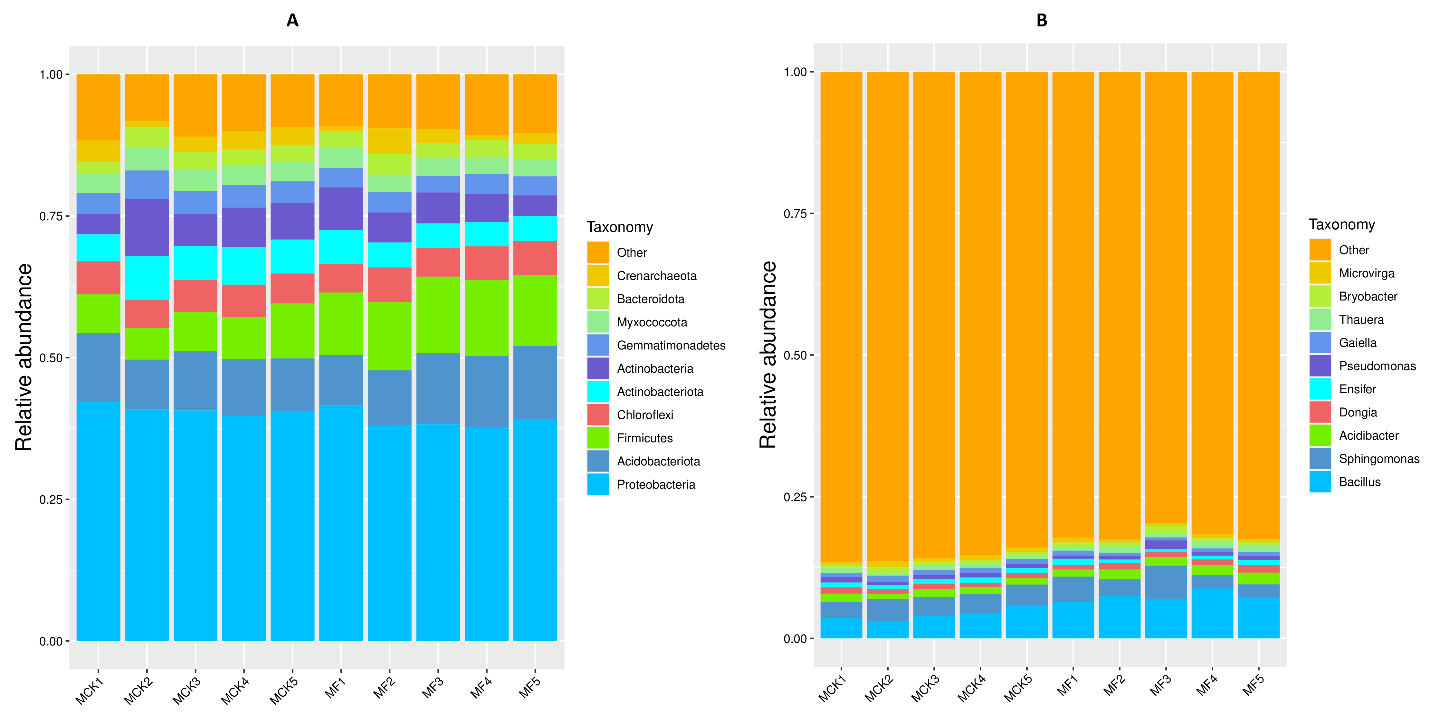
Supplementary Figure S3A,B.** Relative abundance histogram of the top ten active bacterial taxa at the phylum level **(A)** and genus level **(B)** in the rhizosphere of *Cucumis melo var. saccharinus* treatment groups MCK and MF.

**
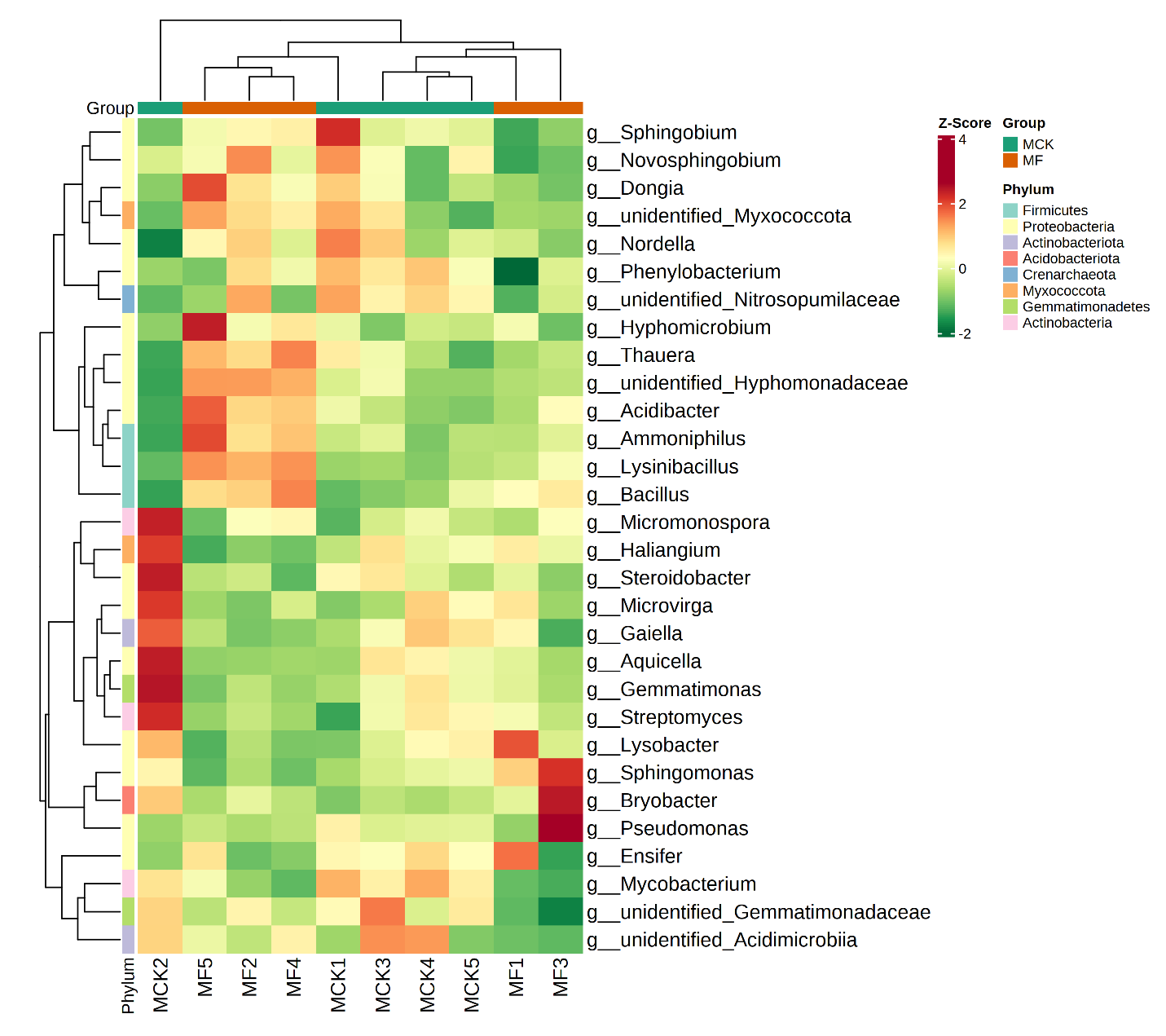
Supplementary Figure S4**. Heatmap analysis showing the dynamic changes in relative enrichment of the top 30 bacterial genera of *Cucumis melo var. saccharinus* treatment groups MCK and MF. Heatmap is based on color-coded Z-scores.

**
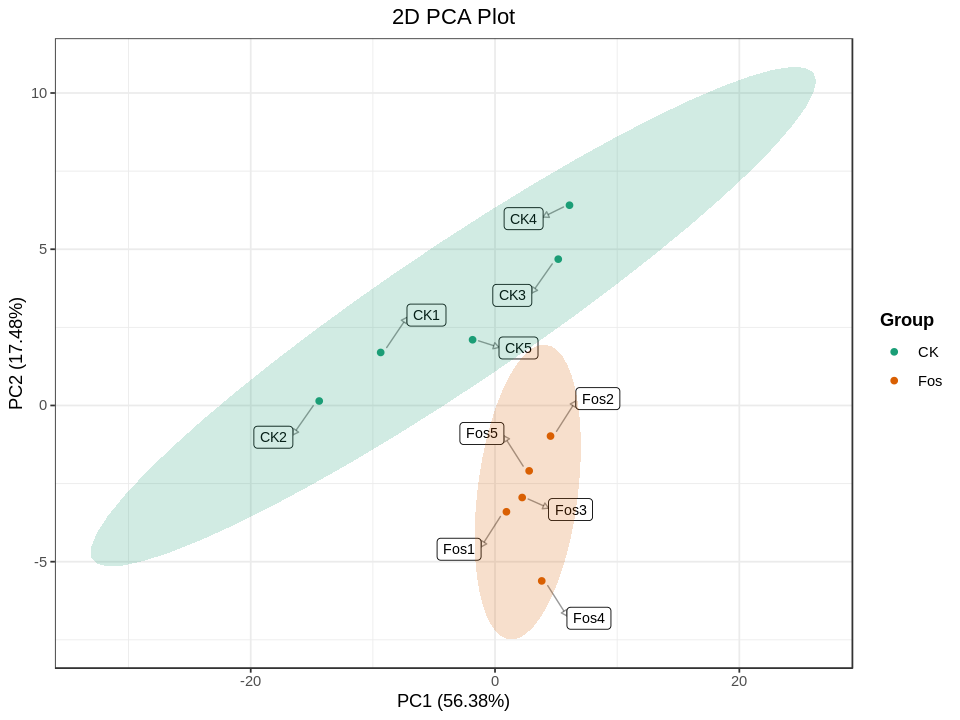
**

**Figure S5.** Principle component analysis of differential metabolites in the rhizosphere microbiome of control group samples (CK1, CK2, CK3, CK4, and CK5) and treatment group samples (Fos1, Fos2, Fos3, Fos4, and Fos5).


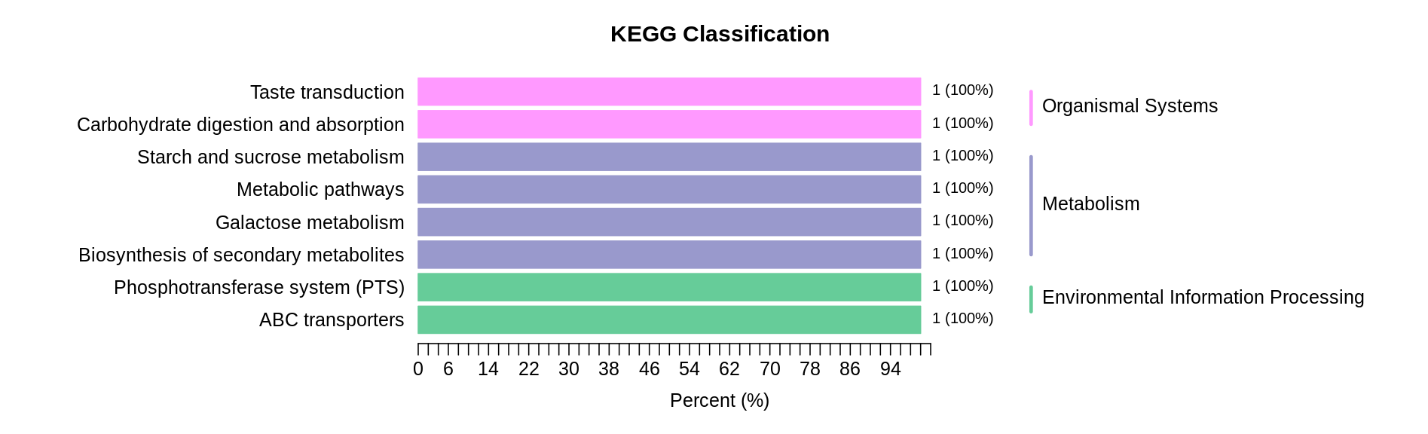
**Supplementary** **Figure S6.** KEGG database analysis showing the highly functionally enriched metabolite pathways of CK vs Fos.
